# Timing of Mental Health Diagnosis in Older Immigrants and Non-Immigrants: Evidence from SHARE

**DOI:** 10.1093/geroni/igaf122.3674

**Published:** 2025-12-31

**Authors:** Ana C Teixeira-Santos, Joana Carvalheiro, Anja Leist

**Affiliations:** Institute for Research on Socio-Economic Inequality, Department of Social Sciences, University of Luxembourg, Esch-sur-Alzette, Diekirch, Luxembourg; University of Glasgow, School of Psychology and Neuroscience, University of Glasgow, Scotland, United Kingdom; University of Luxembourg, Esch-sur-Alzette, Grevenmacher, Luxembourg

## Abstract

The risk of mental health problems is high in older adults. Older immigrants may be at even higher risk due to social and socioeconomic disparities. However, little is known about the timing of their first diagnosis compared to native-born peers. This study examined whether older immigrants have a higher risk of earlier mental health diagnosis compared to non-immigrants and the contribution of social determinants. Data came from SHARE waves 5–9, including 48,154 participants (55.1% women) aged 55+ with no prior mental health diagnosis. The outcome was first self-reported diagnosis of affective or emotional disorders. Stepwise hierarchical Cox mixed-effects models with country-level random intercepts sequentially adjusted for sociodemographic, socioeconomic, family, community engagement, and health care satisfaction factors. In the basic model, migrant status was associated with an 18% increased hazard of diagnosis (HR = 1.18, 95%CI: 1.08–1.29). All included covariates were statistically significant (p < .002). After full adjustment, the HR decreased to 1.16 (95%CI: 1.05–1.27), suggesting that community engagement and health care satisfaction partially account for the higher risk among migrants. Individuals from low-income countries showed a higher risk (HR > 1.30, p < .08) and substantial between-country variation was identified (p = .02). These results suggest that older immigrants were diagnosed earlier, with factors such as social engagement and satisfaction with health care partly explaining this difference, highlighting the importance of culturally sensitive interventions that enhance engagement and participation, as well as policy measures to reduce disparities in mental health outcomes among aging immigrant populations across Europe.

